# Comparative Analyses of *Euonymus* Chloroplast Genomes: Genetic Structure, Screening for Loci With Suitable Polymorphism, Positive Selection Genes, and Phylogenetic Relationships Within Celastrineae

**DOI:** 10.3389/fpls.2020.593984

**Published:** 2021-02-11

**Authors:** Yongtan Li, Yan Dong, Yichao Liu, Xiaoyue Yu, Minsheng Yang, Yinran Huang

**Affiliations:** ^1^Forest Department, Forestry College, Hebei Agricultural University, Baoding, China; ^2^Hebei Key Laboratory for Tree Genetic Resources and Forest Protection, Baoding, China; ^3^Institute of Landscaping, Hebei Academic of Forestry and Grassland, Shijiazhuang, China

**Keywords:** *Euonymus*, chloroplast genome, adaptive evolution, molecular marker, phylograms

## Abstract

In this study, we assembled and annotated the chloroplast (cp) genome of the *Euonymus* species *Euonymus fortunei*, *Euonymus phellomanus*, and *Euonymus maackii*, and performed a series of analyses to investigate gene structure, GC content, sequence alignment, and nucleic acid diversity, with the objectives of identifying positive selection genes and understanding evolutionary relationships. The results indicated that the *Euonymus* cp genome was 156,860–157,611bp in length and exhibited a typical circular tetrad structure. Similar to the majority of angiosperm chloroplast genomes, the results yielded a large single-copy region (LSC) (85,826–86,299bp) and a small single-copy region (SSC) (18,319–18,536bp), separated by a pair of sequences (IRA and IRB; 26,341–26,700bp) with the same encoding but in opposite directions. The chloroplast genome was annotated to 130–131 genes, including 85–86 protein coding genes, 37 tRNA genes, and eight rRNA genes, with GC contents of 37.26–37.31%. The GC content was variable among regions and was highest in the inverted repeat (IR) region. The IR boundary of *Euonymus* happened expanding resulting that the *rps19* entered into IR region and doubled completely. Such fluctuations at the border positions might be helpful in determining evolutionary relationships among *Euonymus*. The simple-sequence repeats (SSRs) of *Euonymus* species were composed primarily of single nucleotides (A)n and (T)n, and were mostly 10–12bp in length, with an obvious A/T bias. We identified several loci with suitable polymorphism with the potential use as molecular markers for inferring the phylogeny within the genus *Euonymus*. Signatures of positive selection were seen in *rpoB* protein encoding genes. Based on data from the whole chloroplast genome, common single copy genes, and the LSC, SSC, and IR regions, we constructed an evolutionary tree of *Euonymus* and related species, the results of which were consistent with traditional taxonomic classifications. It showed that *E. fortunei* sister to the *Euonymus japonicus*, whereby *E. maackii* appeared as sister to *Euonymus hamiltonianus*. Our study provides important genetic information to support further investigations into the phylogenetic development and adaptive evolution of *Euonymus* species.

## Introduction

Chloroplasts (cps) are ubiquitous in plants and originate from symbiotic cyanobacteria (Jin and Daniell, 2015; Gao et al., 2019) with independent genomes and evolutionary routes. It plays important roles in energy conversion, photosynthesis, and the synthesis of fatty acids, chlorophyll, carotene, amino acids, starch, and other compounds (Neuhaus and Emes, 2000; Jensen, 2013). Plant photosynthesis is strictly controlled by heredity, so understanding the gene function and phylogenetic relationships of cp genomes is critical to understanding the origin and evolution of organelles, and has applications in crop improvement and enhancing photosynthetic efficiency (Zhao et al., 2019). The cp genome is mostly a quadripartite structure comprising one large single-copy region (LSC), one small single-copy region (SSC), and two reverse repeats (Abdullah et al., 2019a; Mehmood et al., 2020). However, linear cp genomes have been reported (Oldenburg and Bendich, 2015). Despite the cp genome is relatively conservative in terms of structure, gene order, and gene content (Shahzadi et al., 2019). Many mutational events often occur in cp genomes, including indels, substitutions, inversion, contraction, and expansion of inverted repeats and its effect on the number of genes such as gene loss, duplication, and pseudogenes (Abdullah et al., 2020a; Henriquez et al., 2020). Moreover, Sequence rearrangements have also been reported from various kinds of plants (Sun et al., 2017; Liu et al., 2018).

The *Euonymus* genus belonging to family Celastraceae comprises 220 species, including approximately 111 that occur in China (Lin et al., 2009; Duan and Zhang, 2019; Song et al., 2019a). Species in this genus exhibit rapid growth, tolerance of various light conditions, extreme pruning resistance, resistance to cold and salt, and high resistance to harmful gases, and have the capacity to improve soil and ecological conditions (Chen et al., 2015; Song et al., 2019b). *Euonymus* trees are characterized by attractive shapes and autumn foliage, brightly-colored fruits, and distinctive, winged branches, making them ideal ornamental plants. The morphologically diverse of *Euonymus* species make themselves to different horticultural applications. For example, *Euonymus* species can be planted alone or in rows, as greenbelts, hedgerows, or potted ornamental plants, and can be planted with other tree species; as a result, they are widely used in landscaping in both private gardens and public green spaces. The study of *Euonymus* cp genome is conducive to interspecific relationships, species identification research, plant breeding, resource conservation, development of molecular markers for DNA barcoding, and studies of phylogenetic evolution in *Euonymus* (Huang et al., 2014; Daniell et al., 2016; Zhang et al., 2017). It provides some reference value to make better use of them. Meanwhile, comparative analyses based on cp genome data can provide a more comprehensive interpretation of phylogenetic relationships than using only one or a few DNA fragments (Ruhfel et al., 2014). However, just a few cp genome of *Euonymus* species was sequenced at present. So we need to require more cp genome of *Euoymus* species resolving the phylogenetic relationships among *Euonymus*.

In this study, we sequenced, assembled and annotated the cp genomes of *Euonymus fortunei*, *Euonymus phellomanus*, and *Euonymus maackii*, and compared their sequences with related species including three *Euonymus* species and one *Catha* species from the NCBI. The objectives of this study were to provide whole chloroplast genome data for the three *Euonymus* species; to compare the genomic structure and sequence variation of the chloroplast genome among *Euonymus* species; to identify loci with suitable polymorphism for use in *Euonymus* species identification and phylogenetic studies; to identify positive selection genes as genes potentially contributing to the adaptive evolution of Celastrineae species; and to use data from various sources to construct an evolutionary tree elucidating the phylogenetic relationships in the genus *Euonymus*.

## Materials and Methods

### Plant Materials

In July 2019, fresh leaves of *E. maackii*, *E. fortunei*, and *E. phellomanus* were collected in Hengshui, Hebei Province, China. Leaves were preserved and sent to Beijing Medical Technology Co., Ltd. for chloroplast genome sequencing. Material from nine other Celastrineae species was obtained from the NCBI (Table 1), including four Celastraceae species (*Euonymus japonicus*, *Euonymus hamiltonianus*, *Euonymus schensianus*, and *Catha edulis*), three Ilexaceae species (*Ilex paraguariensis*, *Ilex cornuta*, and *Ilex integra*), one Pentaphylacaceae species (*Pentaphylax euryoides*), and one Staphyleaceae species (*Tapiscia sinensis*) for structural comparison and systematic genomic analysis. Moreover, the complete chloroplast genomes of *Ampelopteris elegans* was also obtained as outgroup.

**Table 1:** Chloroplast (cp) genomes of species along with their NCBI accessions numbers used in the analysis.

S. No.	Species	Accession	References
1	*Euonymus japonicus*	NC_028067	Choi and Park (2015)
2	*Euonymus hamiltonianus*	NC_037518	Lee et al. (2019)
3	*Euonymus schensianus*	NC_036019	Wang et al. (2017)
4	*Catha edulis*	KT_861471	Gu et al. (2018)
5	*Ilex paraguariensis*	NC_031207	Cascales et al. (2017)
6	*Ilex cornuta*	NC_044416	Park et al. (2019a)
7	*Ilex integra*	NC_044417	Park et al. (2019b)
8	*Pentaphylax euryoides*	NC_035710	Yu et al. (2017b)
9	*Tapiscia sinensis*	NC_036960	Ren et al. (2017)
10	*Ampelopteris elegans*	NC_035835	Wei et al. (2017)

### Sequencing, Genome Assembly, and Annotation

Total DNA of fresh young leaves was extracted using a plant DNA extraction kit (TIANGEN Biotech, Beijing, China). Based on the quality, integrity, and concentration of the extracted DNA, the Illumina HiSeq PE150 double-end sequencing strategy was used to build the library. Then FastQC was used to evaluate raw read quality and then raw reads were filtered by removing low-quality reads at the cutoff of Q20 using Trimmomatic (Bolger et al., 2014) to obtain clean reads. GetOrganelle^1^ was used to assemble the plastid genome sequence by selecting 15 million reads from the dataset of clean reads. Both our newly acquired plastid genomes and the downloaded plastid genomes from NCBI website were annotated using the online annotation tool GeSeq (Tillich et al., 2017). All the annotations were manually curated. In addition, we used HMMER (Wheeler and Eddy, 2013) and ARAGORN Version 1.2.38 (Laslett and Canback, 2004) to ensure the prediction accuracy of the encoded protein and RNA genes, respectively. Finally, the resulting plastid genome maps were drawn with Chloroplot (Zheng et al., 2020).

### Indices of Codon Usage

CodonW 1.4.4^2^ (Peden, 2000) was used to evaluate gene codon usage. Five indices, namely, the codon adaptation index (CAI), codon bias index (CBI), optimal codon frequency (FOP), GC content (GC3s), and effective codon number (ENC), were used to evaluate codon preference.

### SSRs and Repeat Sequences Analysis

Simple-sequence repeats were analyzed using MISA (Thiel et al., 2003), with parameters set to 10, 5, 4, 3, 3, and 3 for mono-, di-, tri-, tetra-, penta-, and hexa-nucleotides, respectively. REPuter software (Kurtz et al., 2001) was used to identify forward (F), reverse (R), palindrome (P), and complementary (C) repeats in Celastraceae species that met the requirements of a minimum repeat size of 30bp and 90% or greater sequence identity (Hamming Distance = 3). Tandem Repeats Finder Version 4.04 (Benson, 1999) was used to detect tandem repeats, with parameters set to two for the alignment parameter match and seven for mismatches and indels.

### Comparative Analysis of cp Genomes

The mVISTA program in LAGAN mode^3^ was used to compare the six *Euonymus* cp genomes using the *E. phellomanus* cp genome as a reference. DnaSP version 5.1 (Librado and Rozas, 2009) was used to calculate nucleotide variability (Pi) of the LSC, SSC, and IR regions among the six *Euonymus* species and loci with suitable polymorphism were identified for evolutionary analysis. The step size was set to 200bp and window length to 300bp. MUMmer 4.0 (Kurtz et al., 2004) was used for dot plot analysis. And IRscope (Amiryousefi et al., 2018) was used for the analyses of inverted repeat (IR) region contraction and expansion at the junctions of chloroplast genomes. Gene rearrangements were also observed based on collinear blocks using Geneious R8.1(Kearse et al., 2012) integrated Mauve alignment (Darling et al., 2004).

### Ka/Ks and Positive Selection Analyses

To assess the impact of environmental pressures on the evolution of Celastrineae plants, we calculated the Ka/Ks ratios of the common single copy genes of all species. MAFFT Version 7.453 (Katoh and Standley, 2013) was used to perform multiple sequence alignments of the amino acid sequences of 60 single genes. Pal2nal Version 14 (Suyama et al., 2006) was used to convert amino acid sequence alignment results into nucleic acid alignments. We then combined all alignment results together and used KaKs_Calculator Version 2.0 (Wang et al., 2010) to calculate the Ka and Ks values of SNP differential genes. We used the Optimized Branch-Site model (Yang and Dos, 2010) and the Bayesian Empirical Bayes (BEB; Yang et al., 2005) method to identify genes that were positively selected. TrimAL Version 1.4 (Capellagutiérrez et al., 2009) was used to trim the results of single-gene nucleic acid multiple sequence alignments, and codeml in paml was used for branch-site analysis by calculating the null hypothesis (null model, model = 2, NSsites = 2, Fix-omega = 1, omega = 1) and alternative hypothesis (alternative model, model = 2, NSsites = 2, Fix-omega = 0, omega = 0.2). We ran a Chi Square test in paml Version 4.9 for the LRT test (Yang, 2007), with values of *p* < 0.05 considered indicative of positively selected genes. Finally, the BEB method was used to calculate posterior probabilities of amino acid sites to determine whether sites were positively selected.

### Phylogenomic Analysis

We downloaded the chloroplast genome sequences of the nine aforementioned Celastrineae species from the NCBI, combined them with the three sequenced *Euonymus* species, and conducted a phylogenetic analysis using *A. elegans* as an outgroup. Phylogenetic analysis based on the whole cpDNAs, single copy gene, LSC, SSC and IR were as follows. MAFFT v7.149 (Katoh et al., 2005) was used to align the cpDNAs sequences under default parameters, and the alignment was trimmed by Gblocks_0.91b (Gerard and Jose, 2007) to remove low-quality regions with the parameters: −t = d −b4 = 5 −b5 = h (Castresana, 2000). Nucleotide substitution model selection was estimated with jModelTest 2.1.10 (Darriba et al., 2012) and Smart Model Selection in PhyML 3.0 (Guindon et al., 2010). Then the best fitting GTR+I+G model was selected. As far as the orthologs gene families, they were identified by ORTHOMCL v2.0 program (Li et al., 2003; reciprocal all-by-all BLASTP analysis) with an E-value of 10^−5^. Multiple alignments were generated with the MUSCLE v3.8.31 program (Edgar, 2004), and the alignments were examined visually. The best fitting LG+I+G+F model was determined. Finally, the Maximum-likelihood (ML) methods with 1,000 bootstrap replicates to calculate the bootstrap values were performed for all phylogenetic analyses using PhyML 3.0 and the results were treated with iTOL 3.4.3 (Letunic and Bork, 2016).

## Results and Analysis

### Features of the Chloroplast Genome

**Figure 1:**
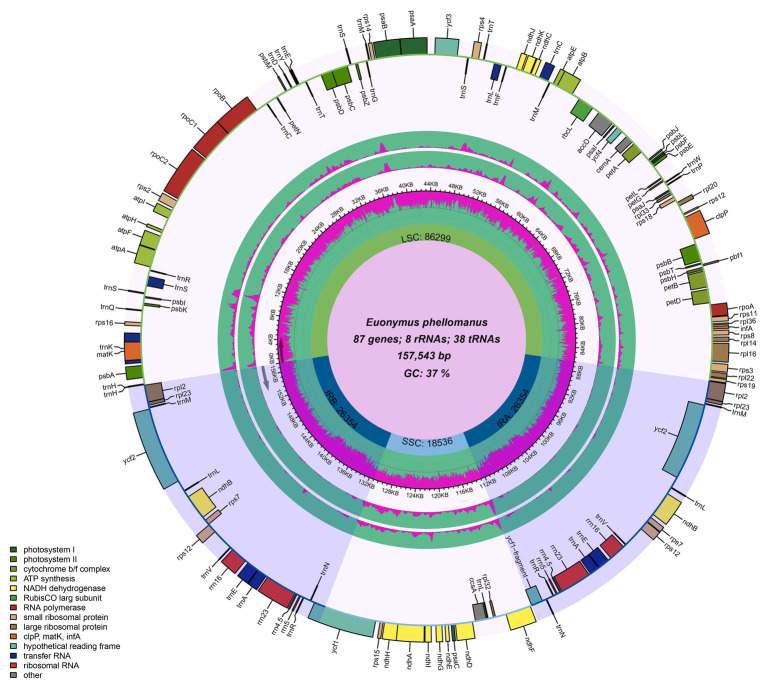
Chloroplast genome maps of *Euonymus* species. The species name and specific information regarding the genome (length, GC content, and the number of genes) are depicted in the center of the plot. Extending outward, the middle two layers are the nucleotide diversity of *E.fortunei* (inner) and *E. maackii* (Outer) compared with *E. phellomanus*, respectively.

**Figure 2:**
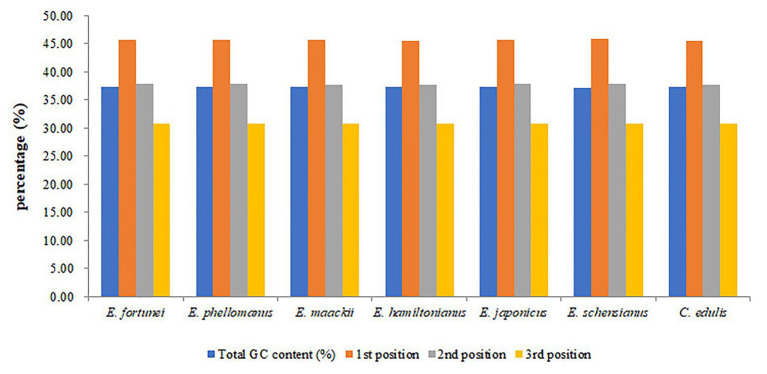
The GC (%) composition in different positions of coding sequence (CDS) region of species within Celastraceae.

The chloroplast genome of the sequenced *Euonymus* species comprised a typical covalently closed, double-stranded circular molecule without the large fragment missing (Figure 1). Dot plot analysis indicated that genome content and structure were similar among *Euonymus* species, and no substantial rearrangement was detected (Supplementary Figure S1). And the chloroplast genomes of *Euonymus* species revealed similarity and formed similar collinear blocks (Supplementary Figure S2). The complete chloroplast genomes of the three species of *Euonymus* ranged from 156,860 (*E. maackii*) to 157,611bp (*E. fortunei*), with 37.26–37.31% GC content (Table 2). It had a typical circular structure including a LSC region of 85,826–86,299bp, a SSC region of 18,319–18,536bp, and a pair of IRs (IRa, IRb) each 26,341–26,700bp (Table 2; Figure 1). Besides, the length of the coding region ranged from 78,552 (*E. fortunei*) to 79,239bp (*E. maackii*) and the length of the non-coding region ranged from 77,621 (*E. maackii*) to 79,059bp (*E. fortunei*). A total of 130–131 chloroplast genes, comprising 85–86 protein coding genes, 37 tRNA genes, and eight rRNA genes were detected. The GC content of the chloroplast genome differed among locations and among genes coding for different functions. The gene coding region (38.14–38.15%) had significantly higher GC content than the non-coding region (36.40–36.48%). Moreover, GC content was highest in the IR region (42.66–42.71%), followed by the LSC region (35.08–35.20%) and the SSC region (31.74–31.78%). The rRNA genes had the highest GC content of the entire coding region (55.36–55.40%). The total GC content (37.26–37.31%) was lower than in the IR region, but higher than in the SSC and LSC regions. And the GC% content of the first position was higher compared to those of the second and third positions (Figure 2). A total of 16 genes harbored introns, of which *clpP* and *ycf3* contained two introns (Supplementary Table S1).

**Table 2:** The basic characteristics of the chloroplast genomes of six *Euonymus* species and *C. edulis*.

	*E. fortunei*	*E. phellomanus*	*E. maackii*	*E. hamiltonianus*	*E. japonicus*	*E. schensianus*	*C. edulis*
Total length (bp)	157,611	157,543	156,860	157,360	157,637	157,702	157,960
Total GC(%)	37.26	37.30	37.31	37.25	37.26	37.19	37.28
LSC length (bp)	85,892	86,299	85,826	86,399	85,941	86,026	86,315
GC (%)	35.08	35.18	35.20	35.10	35.08	35.02	35.11
SSC length (bp)	18,319	18,536	18,352	18,317	18,340	18,528	18,485
GC content (%)	31.78	31.78	31.74	31.70	31.79	31.64	31.82
IR length (bp)	26,700	26,354	26,341	26,322	26,678	26,574	26,580
GC content (%)	42.66	42.71	42.71	42.72	42.67	42.68	42.70
Coding region length (bp)	78,552	79,005	79,239	79,968	78,540	78,069	79,227
Coding region GC (%)	38.15	38.15	38.14	38.03	38.15	39.78	38.01
Noncoding region length (bp)	79,059	78,538	77,621	77,392	79,097	79,633	78,733
Noncoding region GC (%)	36.40	36.44	36.48	36.44	36.38	36.21	36.55
Protein-coding gene num	86	85	86	86	86	86	86
Protein-coding region GC (%)	38.15	38.15	38.14	38.03	38.15	38.19	38.01
Intron GC (%)	34.34	34.52	34.5	34.69	34.35	34.17	34.23
rRNA GC (%)	55.38	55.40	55.36	55.36	55.38	55.38	55.25
tRNA GC (%)	53.03	52.99	53.17	53.13	53.03	53.10	53.05
Total tRNA	37	37	37	37	37	37	37
Total rRNA	8	8	8	8	8	8	8
Total gene num	131	130	131	131	131	131	131

**Figure 3:**
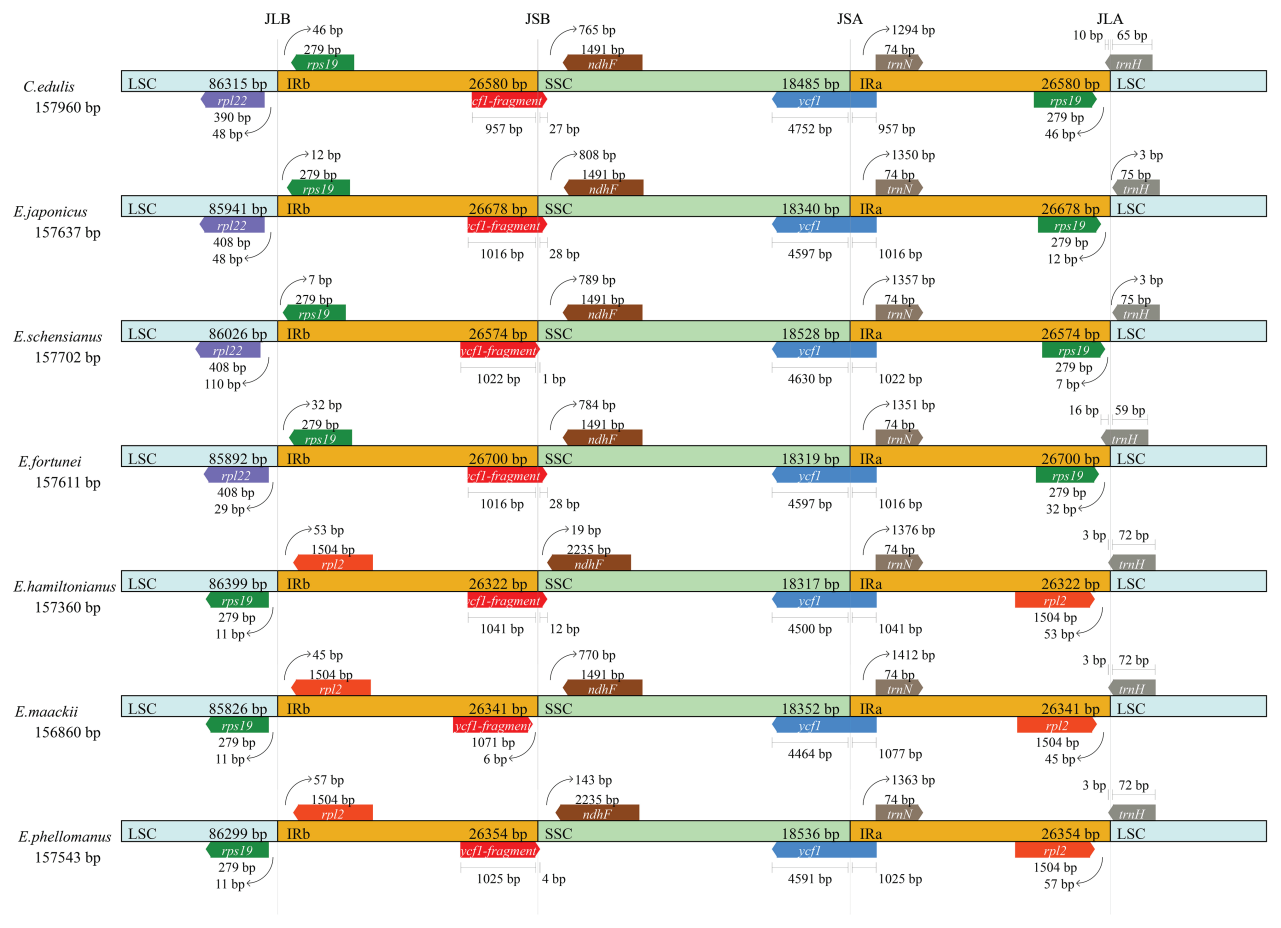
Comparison of the borders of large single-copy (LSC), small single-copy (SSC), and inverted repeat (IR) regions among seven Celastraceae cp genome.

Contraction and expansion of the IR region is common, a phenomenon known as ebb and flow (Goulding et al., 1996). We compared the JL (LSC/IR) and JS (IR/SSC) border positions of the *Euonymus* chloroplast genome (Figure 3). The length of the IR regions was similar, ranging from 26,322 to 26,700bp, with some expansion. Some notable differences were found at the junctions of JLB (IRb/LSC) and JLA (IRa/LSC) among the species. The JLB junction point of *C. edulis*, *E. japonicus*, *E. schensianus*, and *E. fortunei* was located between the *rpl22* and *rps19*, and the length of the *rps19* in IRb from the JLB was 7–46bp. However, the *rps19* of *E. hamiltonianus*, *E. maackii*, and *E. phellomanus* were located in the LSC completely. What is more, the *trnH-GUG* and *rps19* among *C. edulis*, *E. japonicus*, *E. schensianus*, and *E. fortunei* was located at JLA junction. Among them, *C. edulis* and *E. fortunei* showed integration of *trnH-GUG* into the IRa region 10 and 16bp, respectively. While the *trnH-GUG* of *E. schensianus* and *E. japonicas* was completely found in the LSC region. The JLA of *E. hamiltonianus*, *E. maackii*, and *E. phellomanus* was located on the right side of the *rpl2* and the *trnH-GUG* extended into the IRa with the length of 3bp. Furthermore, the *ycf1* located on the JSB (IRb/SSC) were detected as pseudogenes in all species. Detail of IR contraction and expansion has been provided in Figure 3.

### Indices of Codon Usage

**Figure 4:**
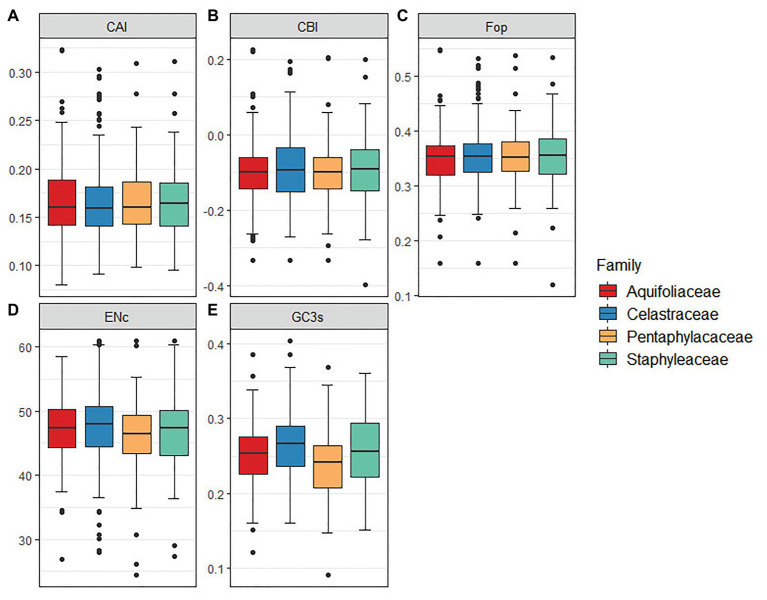
The comparative analysis of codon usage bias in 12 Celastrineae species. **(A)** Codon adaptation index (CAI), **(B)** Codon bias index (CBI), **(C)** Frequency of optimal codons index (Fop), **(D)** Effective number of codons (ENc). **(E)** GC of synonymous codons in third position (GC3s).

The results indicated that CAI, CBI, and FOP values were similar among Celastrineae species, while ENC and GC3s values were slightly higher in Celastraceae than in other families (Figure 4).

### Repeat Sequences Analysis of Celastraceae

**Figure 5:**
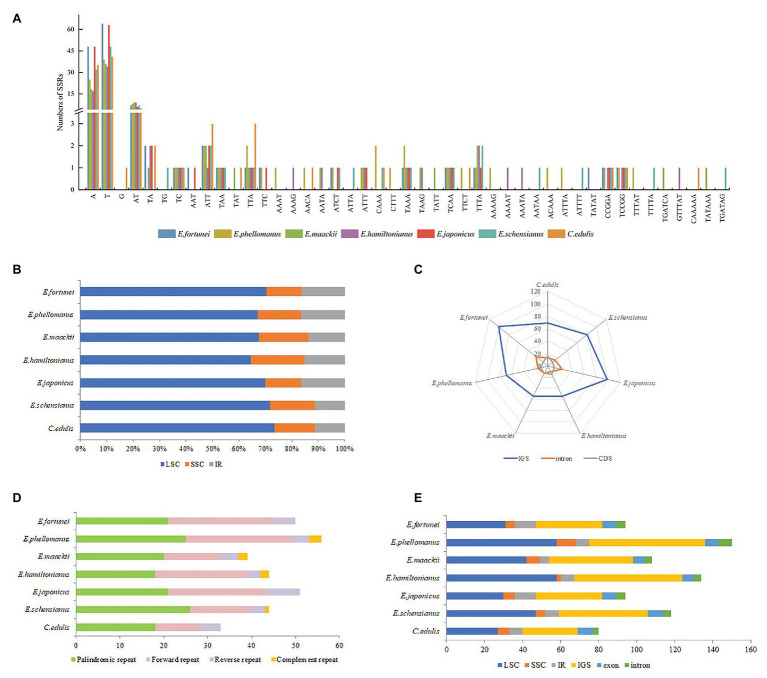
**(A)** Analysis of simple-sequence repeats (SSRs) in the chloroplast genomes of seven Celastraceae species; **(B)** Frequency of SSRs in the LSC, IR, SSC region; **(C)** Frequency of SSRs in the intergenic regions, protein-coding genes, and introns; **(D)** Number of Palindromic repeat, Direct repeat, Reverse repeat, Complement repeat; **(E)** Distribution of tandem repeats in genomic regions and exon, intergenic spacer (IGS), and intron regions.

The high rate of polymorphism in SSRs at the species level makes them one of the most common molecular markers in phylogenetic and population genetics studies. In total, 79 (*E. hamiltonianus*) to 135 (*E. fortunei*) SSRs were detected in the chloroplast genome of the Celastraceae species, the majority of which were mononucleotide repeats (51–112), followed by dinucleotides (8–12), tetranucleotide (3–12), trinucleotides (3–7), pentucleotide (2–6), and hexnucleotide (1–2; Figure 5A). Mononucleotide nucleotide repeats may play a more important role in genetic variation than other types of SSRs. SSRs were mainly composed of the single nucleotides (A)n and (T)n, and their lengths were mostly in the 10–12bp range. Aside from the presence of a G in the SSRs of *C. edulis*, the remainder were composed of A or T only, indicating that the base composition of SSRs was biased toward the use of A/T bases. Moreover, SSRs of the chloroplast genome of Celastraceae species were primarily distributed in the LSC and SSC regions (Figure 5B), and these two regions were also the main distribution regions of a few genes in the chloroplast genome. In addition, the analysis of SSR locations revealed that most SSRs were distributed in the non-coding regions of the genome, namely the intergenic and intron regions (Figure 5C).

Long repetitive sequences with a length ≥30bp may promote rearrangement of the chloroplast genome and increase the function of species genetic diversity (Qian et al., 2013). In total, 33 (*C. edulis*) to 56 (*E. phellomanus*) long repeat sequences were predicted in the chloroplast genome of the Celastraceae species, including 18–26 palindromic repeats, 10–24 direct repeats, 3–8 reverse repeats, and 1–3 complement repeats. Of these, palindromic, forward and reverse repeats were common to seven species, while complement repeats were detected only in *E. phellomanus* (3), *E. hamiltonianus* (2), *E. maackii* (2), and *E. schensianus* (1; Figure 5D). In addition, 40 (*C. edulis*) to 75 (*E. phellomanus*) tandem repeats were detected with lengths mostly in the range 25–109bp. These tandem repeats were mainly distributed in the LSC and non-coding regions (Figure 5E).

### Comparative Genomic Analysis and Suitable Polymorphic Loci Identification

**Figure 6:**
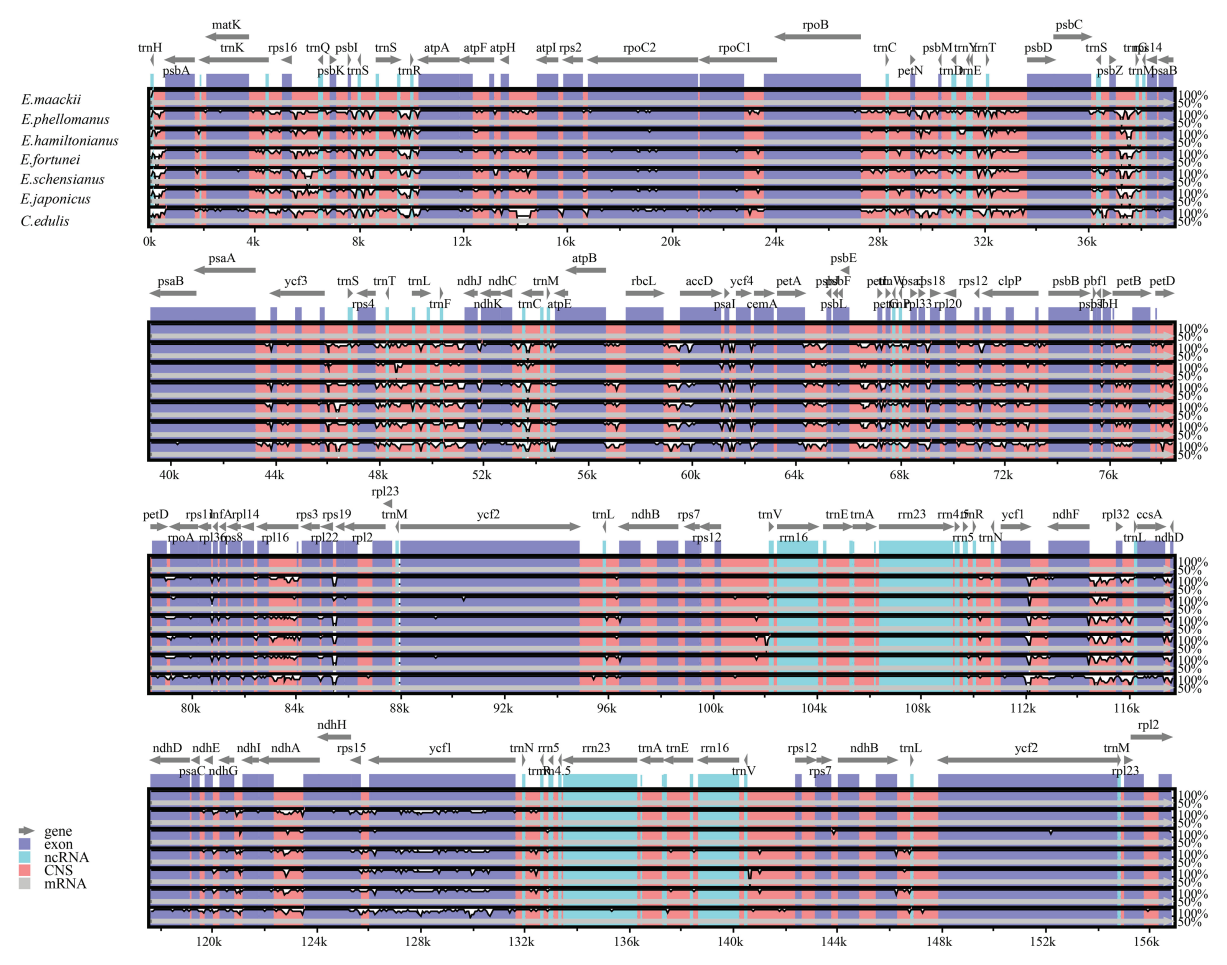
Comparison of six *Euonymus* cp genomes using mVISTA, with the *E. phellomanus* genome as the reference. The *y*-axis represents the percent identity within 50–100%. Gray arrows indicate the direction of gene transcription. Blue blocks indicate conserved genes, while red blocks indicate conserved noncoding sequences (CNS).

Pairwise determination of divergent regions was conducted by mVISTA among *Euonymus* using *E. phellomanus* sequence as a reference (Figure 6). The results indicated that the six *Euonymus* cp genomes were relatively conserved and similar. In general, the LSC and SSC regions exhibited greater variation than did the IR region and variation was greater in the non-coding region than in the coding region. Studies of the genetic diversity and evolution of Celastrineae species using non-coding cpDNA sequences are lacking; it is therefore important to identify suitable polymorphic genes to investigate further the systematic evolution and biogeographic relationships of this group.

**Figure 7:**
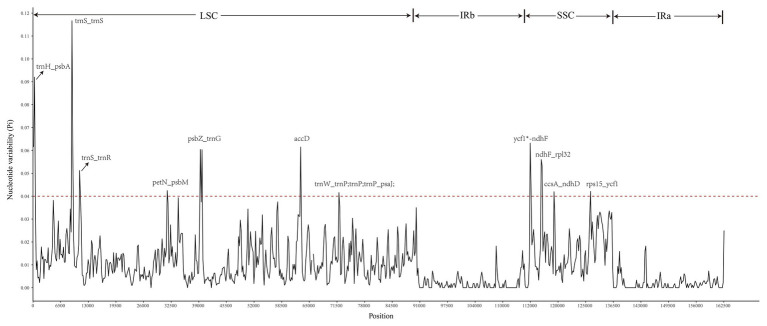
Comparison of nucleotide diversity (Pi) values among the six *Euonymus* species (window length: 300bp, step size: 200bp). *X*-axis, position of the midpoint of each window; *Y*-axis, nucleotide diversity (pi) of each window.

A sliding window analysis indicated that most of the variation in the cp genomes of the six *Euonymus* species occurred in the LSC and SSC regions (Figure 7). The average nucleotide differences of intergenic spacer (IGS) regions were found the highest. The most divergent non-coding regions were *trnH/psbA*, *trnS/trnS*, *trnS/trnR*, *petN/psbM*, *psbZ/trnG*, *trnW/trnP*; *trnP*; *trnP/psaJ*, ycf1*/ndhF, *ndhF/rpl32*, *ccsA/ndhD*, and *rps15/ycf1* (Pi > 2.0; Table 3). The protein-coding regions of *accD* were also included in the suitable polymorphic loci. Although coding regions were conserved in these cp genomes, sequence variation was observed among the six cp genomes in the *ycf1*, *ndhF*, and *rpoC2* gene. These polymorphic loci might be helpful for phylogenetic inference and population genetic studies of the species of genus *Euonymus*.

**Table 3:** Polymorphic loci identified by comparing six *Euonymus* species.

S. No.	Start	End	Pi	Gene
1	1	476	0.0920188	*trnH/psbA*
2	8,415	8,654	0.1166667	*trnS/trnS*
3	9,865	10,114	0.0512438	*trnS/trnR*
4	29,968	30,241	0.0424731	*petN/psbM*
5	37,412	37,898	0.0604167	*psbZ/trnG*
6	59,812	60,057	0.0614916	*accD*
7	68,208	68,500	0.0415771	*trnW/rnP; trnP; trnP/psaJ;*
8	112,478	112,961	0.0632184	ycf1^*^/ndhF
9	115,054	115,451	0.0560137	*ndhF/rpl32*
10	117,751	118,035	0.0419655	*ccsA/ndhD*
11	126,222	126,496	0.0421314	*rps15/ycf1*

### Ka/Ks Ratios of Species Pairwise and Positive Selection Analyses

**Figure 8:**
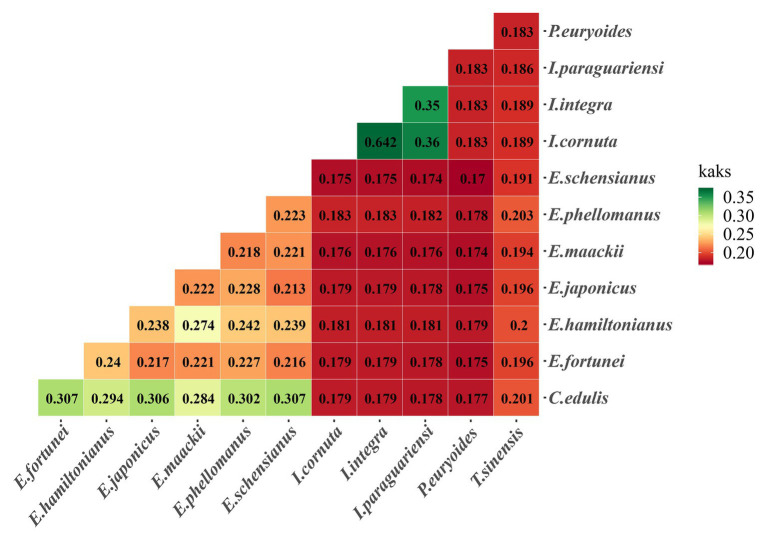
Pairwise Ka/Ks ratios 12 Celastrineae species. This heatmap shows pairwise Ka/Ks ratios between every sequence in the multigene nucleotide alignment.

Ka/Ks ratios provide information on the effects of selection pressures on individual sequences. The two *Ilex* species had higher Ka/Ks ratios compared to other species. The highest overall value was detected in one of the *Ilex* species, followed by the Celastraceae species (Figure 8).

Sixty common single-copy CDS genes from 12 Celastrineae species were subjected to positive selection analyses (Supplementary Table S2). The *p*-values of the protein coding genes *rpoB* were <0.05, indicating positive selection.

### Chloroplast Phylogenetic Analysis

**Figure 9:**
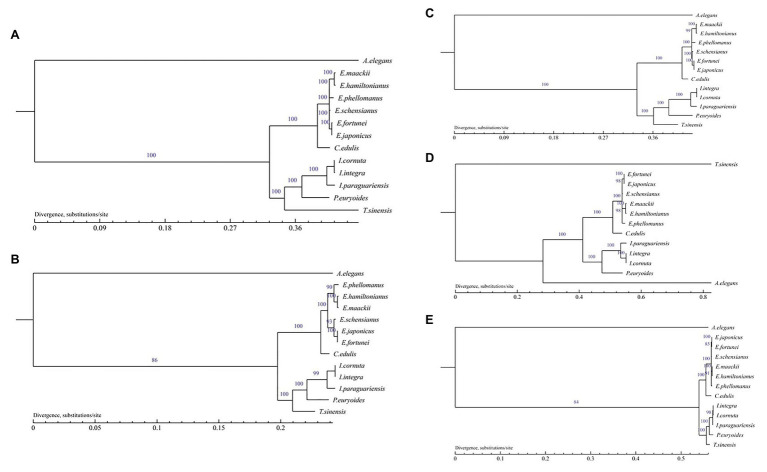
Phylogenetic trees of the *Euonymus* species based on the chloroplast genome by MP. **(A)** Phylogenetic tree constructed using the complete chloroplast genome data. **(B)** Phylogenetic tree constructed using single copy genes; **(C)** Phylogenetic tree constructed using LSC data; **(D)** Phylogenetic tree constructed using SSC data; and **(E)** Phylogenetic tree constructed using IR data.

Phylogenetic analysis with *Euonymus* plastid genomes was performed with the ML method based on the complete chloroplast genomes, single copy gene, LSC, SSC, and IR region, with the outgroup *A. elegans*. The best fit model GTR+I+G and LG+I+G+F of the complete chloroplast genomes, LSC, SSC, IR region and single copy gene were selected, respectively. All phylogenetic trees exhibited similar clustering and a high level of support, and were consistent with traditional taxonomic classifications, except the tree based on SSC. Species within the same genus or family were grouped together (Figure 9). In particular, *E. fortunei* and *E. japonicus* were clustered more closely to one another than to other *Euonymus* species. Moreover, *E. maackii* was found as sister taxa to *E. hamiltonianus*.

## Discussion

### Plastome Features

The structure, gene organization and gene content of the cp genome of *Euonymus* species were highly conserved, which is similar to the other Celastrineae species (Choi and Park, 2015; Cascales et al., 2017; Gu et al., 2018). It exhibited a typical circular tetrad structure and no IR region was completely lost, which had occurred in *Pisum sativum* and *Medicago truncatula* (Saski et al., 2005). The cp genome had the conservative nature regardless of phylogenetic position. When comparing the families that had the different diverged up time, such as the *Ginkgo* (Yang et al., 2020), *Magnolia* (Sima et al., 2020), *Abies* (Su et al., 2019), *Nymphaea* (Kim et al., 2019), and *Pyrus* (Li et al., 2018), we found that they both had conserved cp genome structures in terms of gene content and gene arrangement. Moreover, the Araceae’s plastid genome was also conserved compared with Orchidaceae and Fabaceae that diverged up to 50 million years later from Araceae showing significant gene rearrangements due to various inversion events (Abdullah et al., 2020b).

The total length of the chloroplast genome of *Euonymus* species was 156,860–157,611bp, encoding a total of 130–131 genes, including 85–86 protein coding genes, the same number of tRNA and rRNA genes. GC content plays an important role in genome recognition, and differences in the genomes of different species are apparent through changes in base composition (Zhu et al., 2017). The total GC content of the *Euonymus* species was 37.26–37.31%, well within the usual range for chloroplast genomes of seed plants (34–40%). The GC content was highest in the IR region, mainly owing to the presence of four rRNA genes with high GC content in this region and lowest in the SSC region. The uneven distribution of GC content may be an important factor in the conservatism of the IR region relative to the LSC and SSC regions.

Shrinkage and expansion of the IR boundary is one of the main drivers of changes in the length of the chloroplast genome (He et al., 2017). And it can lead to the loss of one copy of genes, the duplication of genes, or the origination of pseudogenes in the chloroplast genome of angiosperms (Yu et al., 2017a; Abdullah et al., 2019b). (Abdullah et al. 2020a) found that the rate of evolution of protein-coding genes was affected by the contraction and expansion of IRs among subfamily Pothoideae. Here, we compared border regions among the *Euonymus* cp genomes and found that a difference of nearly 378bp of IR region between the smallest (*E.hamiltonianus*) and largest (*E. fortunei*) were detected. The plastomes of *E.fortunei*, *E.japonicus*, and *E.schensianus* showed expansions of the IRs and contractions on the LSC. This resulted that the *rps19* located in LSC of *E. hamiltonianus*, *E. maackii*, and *E. phellomanus* entered into the IRb and doubled completely. The *ycf1* observed at the junction of IRb and SSC in *Euonymus* species were also founded to be pseudogenized. This phenomenon has also been reported in other angiosperms (Yao et al., 2016; Shahzadi et al., 2019). Our study agreed with the study that the IR contraction and expansion might be helpful in the study of evolutionary patterns (Iram et al., 2019).

### Identification of Repeated Sequences

Repeated sequences may have the effect of promoting chloroplast genome rearrangement and recombination (Weng et al., 2013; Zhou et al., 2019). SSRs are widely distributed in the chloroplast genome of eukaryotes, and have the advantages of simple structure, relative conservatism, polymorphism making them efficient molecular markers that are widely used in species identification, analyses of genetic differences among individuals, and population evolution studies (He et al., 2012; Pauwels et al., 2012). In total, 79–135 SSRs were found in the chloroplast genome of Celastraceae species, including mononucleotide repeats, dinucleotides, tetranucleotide, trinucleotides, pentucleotide, and hexnucleotide. Of these, Mononucleotide nucleotides, which were rich in A/T, were most abundant. Our results are consistent with previous reports that SSRs usually consist of polyA or polyT repeats and rarely contain G or C repeats (Kuang et al., 2011; Ye et al., 2018); this may be because A/T change more easily than do G/C. SSRs of the Celastraceae species were distributed mainly in the intergenic regions as compared to the gene regions and introns and were found primarily in the LSC and SSC regions. Genomic evolution studies imply that generation of new genes originates from repetitive sequences. The higher number of SSRs in SSCs may be one reason for the greater variability of the latter, as compared to IR regions (Wolfe et al., 1987). Among *Euonymus* species plastid genome, we also observed abundance of oligonucleotide repeats, which have been suggested as a proxy for identification of polymorphic loci (Ahmed et al., 2012). The oligonucleotide repeats are usually considered to produce substitutions, insertions-deletions (InDels), inversion and rearrangements (Keller et al., 2017). (Abdullah et al. 2020c) research in the eudicot family Malvaceae showed that at family and subfamily level comparisons, 88–96% of the repeats showed co-occurrence with SNPs, whereas at the genus level, 23–86% of the repeats co-occurred with SNPs in same bins. Moreover, Michael (McDonald et al., 2011) found that repeat sequences are closely associated with a large proportion of indels and that the abundance of repeat sequences is linked with regions of increased nucleotide diversity.

### Identification of Suitable Polymorphic Loci

Currently, DNA barcode technology is widely used in species identification, resource management, phylogeny, and evolution (Gregory, 2005; Liu et al., 2019). The comparative genome analysis using mVISTA indicated that the DNA sequence of *Euonymus* species was high level of similarity. Compared with the LSC and SSC regions, the sequence differentiation in the IR region was slower and more conservative due to the replication correction caused by the higher gene conversion between the two IR regions (Khakhlova and Bock, 2006). We also identified some polymorphic regions by comparison of six *Euonymus* species using the sliding window analysis. The nucleotide diversity was higher in SCs and non-coding regions than in IRs and coding regions, which is consistent with findings from other taxa (Ren et al., 2018). The *trnH/psbA*, *trnS/trnS*, *trnS/trnR*, *petN/psbM*, *psbZ/trnG*, *trnW/trnP*; *trnP*; *trnP/psaJ*, ycf1*/ndhF, *ndhF/rpl32*, *ccsA/ndhD*, *rps15/ycf1* and protein-coding gene *accD* were identified as hypervariable loci at the species level within *Euonymus*. Among the most divergent noncoding regions, some were shown in previous studies to be highly variable and of high phylogenetic utility i.e., *trnH-GUG/psbA*, *ndhF/rpl32*, and *petN/psbM* (Shaw et al., 2005; Doorduin et al., 2011; Fonseca and Lohmann, 2017; Thode and Lohmann, 2019). The relatively high divergence observed in the *accD*, *ycf1*, *ndhF*, and *rpoC2* genes is similar to that observed in other angiosperms (Park et al., 2018; Thode and Lohmann, 2019). A evolutionary tree conducted by using *psbA/trnH*, *rp136/infA/rps8*, and *trnC/ycf6* showed that Sect. Echinococcus group and Sect. Kalonymus group were clustered together, but the *Euonymus macroptera* belongs to Sect. Kalonymus was clustered into the Sect. Echinococcus (Li, 2014). In this study, these new identified suitable polymorphic loci can be used to cost effective, develop authentic and robust molecular markers and provide information about the phylogeny of *Euonymus* species.

### Adaptation Evolution of Celastrineae Plastome

Analyzing the adaptive evolution of genes has value for the study of variation in gene functions, structural changes, and the evolutionary trajectory of species (Nei and Kumar, 2000). Synonymous and non-synonymous nucleotide substitution patterns are important markers for gene evolution research (Raman and Park, 2016). Estimates of the ratio of non-synonymous (Ka) to synonymous (Ks) substitution rates can be used as a basis to speculate about selection pressures and the evolutionary tendencies of protein-encoding genes. The Ka/Ks ratio may be equal to, less than, or greater than one, indicating that evolution is subject to either neutral, negative, or positive selection, respectively (Yang and Nielsen, 2002). In this study, we examined the selective pressure of 60 common single copy genes in different branches of Celastrineae to test adaptive genes. The result showed that most protein coding genes were associated with low sequence difference and purification selection, which is consistent with other studies reporting that positive selection is less common than neutral evolution and negative selection (Yin et al., 2018). We also found that the *rpoB* genes were positively selected. The *rpo* genes (*rpoA*, *rpoB*, *rpoC1*, and *rpoC2*) are relatively rapidly evolving regions (Krawczyk and Sawicki, 2013). Among these, the *rpoB* gene within the plastid genome encodes the *β*-subunit of RNA Polymerase which is homologous to its bacterial counterparts (Shinozaki et al., 1986). It is located in the gene cluster *rpoB-rpoCl-rpoC2* related to self-replicating. A research showed that the *rpoB* gene of rice chloroplast RNA polymerase was found to be highly expressed in unexpanded immature leaves that contained proplastids, indicating the specific expression of *rpoB* at an early stage of chloroplast development (Hitoshi et al., 1996). And the *rpoB* gene has been used in phylogeny reconstruction, representing DNA barcodes for land plants (Krawczyk and Sawicki, 2013).

## Data Availability Statement

The original contributions presented in the study are publicly available. This data can be found at: https://www.ncbi.nlm.nih.gov/search/all/?term=MW288090/MW288090, https://www.ncbi.nlm.nih.gov/search/all/?term=MW288091/MW288091, and https://www.ncbi.nlm.nih.gov/search/all/?term=MW288092/MW288092.

## Author Contributions

MY conceived and designed the experiments. YoL, YiL, and XY collected the samples and analyzed the sequence data. YoL and YD drafted the manuscript. YoL, MY, and YH revised the manuscript. All authors read and approved the final manuscript.

### Conflict of Interest

The authors declare that the research was conducted in the absence of any commercial or financial relationships that could be construed as a potential conflict of interest.

## Footnotes

^1^https://github.com/Kinggerm/GetOrganelle

^2^http://codonw.sourceforge.net

^3^http://genome.lbl.gov/vista/mvista/about.shtml
