# Supplementary figures and images for: Comparative Analyses of Euonymus Chloroplast Genomes: Genetic Structure, Screening for Loci With Suitable Polymorphism, Positive Selection Genes, and Phylogenetic Relationships Within Celastrineae
Source: Front Plant Sci. 2021 Feb 11;11:593984. doi: 10.3389/fpls.2020.593984 (PMC7905392; doi:10.3389/fpls.2020.593984)

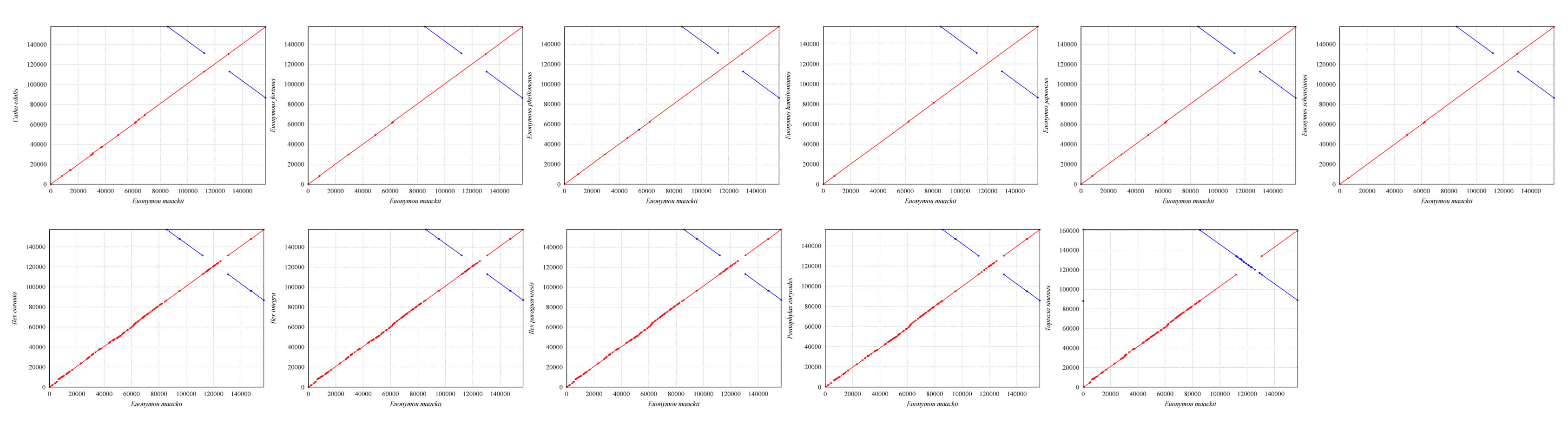

Supplement: Supplementary file 1 [file Data_Sheet_1.zip › Supplementary material/Figure S1.jpg]

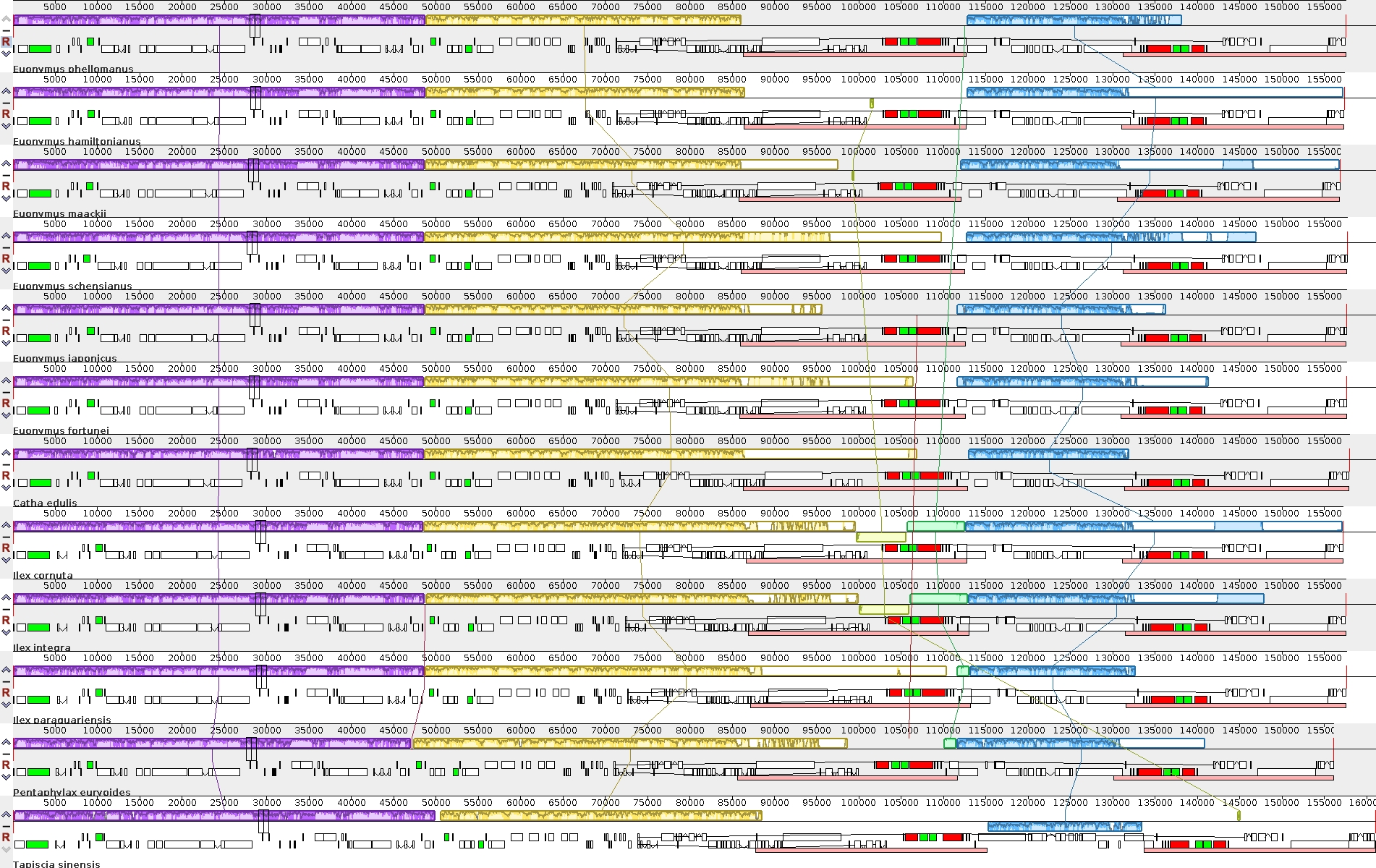

Supplement: Supplementary file 1 [file Data_Sheet_1.zip › Supplementary material/Figure S2.jpg]
